# *Toxocara* (Nematoda: Ascaridida) and Other Soil-Transmitted Helminth Eggs Contaminating Soils in Selected Urban and Rural Areas in the Philippines

**DOI:** 10.1155/2014/386232

**Published:** 2014-10-14

**Authors:** Vachel Gay V. Paller, Emmanuel Ryan C. de Chavez

**Affiliations:** Animal Biology Division, Institute of Biological Sciences, University of the Philippines Los Baňos, 4030 Laguna, Philippines

## Abstract

The extent of contamination of soils with soil transmitted helminthes (STH) eggs, particularly *Toxocara*, was determined in selected urban and rural towns of Laguna, Philippines. Soil samples were collected from public schools, house yards, and empty lots. Results revealed that, of the 1480 soil samples collected, 460 (31%) were positive for STH eggs. *Toxocara* sp. was the most prevalent (77%), followed by *Ascaris* sp. (11%), hookworms/strongyles/free-living nematodes (7%), and *Trichuris* sp. (5%). Some soil physicochemical parameters were also determined and associated with *Toxocara* eggs prevalence and density in soil. Results revealed that *Toxocara* sp. eggs were most prevalent in less acidic, relatively high temperature and high moisture soil conditions. They were also prevalent in sandy, silty, and loamy soil textures but less prevalent in clayey. No significant differences were found between depth 1 (0–5 cm) and depth 2 (6–10 cm). This study revealed that *Toxocara* sp. eggs are ubiquitous and the extent of contamination in soils from the selected towns of Laguna is relatively high. Hence, the data generated in this study can be used in promoting public awareness, particularly for pet owners and local health officials, for effective prevention and control of this parasitosis.

## 1. Introduction

Soil is a potential source of human diseases caused by many helminth parasites. Based on the report of the World Health Organization (WHO), last century two billions of the world populations are infected by soil-transmitted helminthes (STH) such as* Ascaris lumbricoides*, hookworms, and* Trichuris trichiura*. These parasites commonly cause infections and diseases of chronic morbidity and debilitation [1]. However,* Toxocara* sp. is a little-known parasite but is reported to be an emerging STH parasitic infectious agent.

Toxocariasis is a zoonotic disease caused by the larva of* Toxocara canis *and* T. cati* from dogs and cats, respectively. Humans are commonly infected by ingestion of embryonated eggs from the soil or through contaminated hands and fomites. Before becoming infective, the ova undergo further development in the soil and can be viable for several years. Children are the most vulnerable ones because of their habit of playing with soil or objects contaminated with eggs [2].* Toxocara* causes visceral larval migrans characterized by migration of larvae to different organs including the eyes and brain. It can cause stunted growth, decreased physical activity, and poor physical and mental development in children. Moreover, toxocariasis accounts for preventable childhood illnesses and blindness associated with poor hygiene [3].

There is already an evidence of* Toxocara* infection among public school children in Los Baños, Philippines [4]. Through ELISA test, the study reported 43% seroprevalence of* Toxocara* sp. infection among public school children. Soil contamination by parasite eggs was also reported recently in the Philippines but only on a small scale in a small village [5]. No studies have been conducted on a larger scale regarding the extent of* Toxocara *sp. eggs contaminating the soil even though feral dogs and cats abound in the country. Furthermore, current understanding of the health impact of toxocariasis is poor due to insufficient public awareness and lack of interventions of local health units.

Thus, in this research, the extent of soil contamination by* Toxocara* eggs in selected school grounds, house yards, and playgrounds in the four municipalities of Laguna, namely, Los Baños, Bay, Calauan, and Calamba, was investigated. In addition, some physicochemical parameters of soils were determined and correlated with prevalence and density of* Toxocara* sp. eggs. Hence, this study aimed to provide information on the extent of STH contamination in selected towns of Laguna, Philippines, and to ascertain the relationship between prevalence rates with some soil characteristics. The data generated in this study can be used in promoting public awareness to prevent contamination of soils and prevent children from becoming infected with soil-borne parasites.

## 2. Methodology

### 2.1. Collection Sites and Sampling Design

The survey was carried out for a period of two years in the towns of Bay, Los Baños, Calamba, and Calauan, Laguna. The villages from the selected towns were randomly selected from which school grounds, house yards, and empty lots were identified. Thirty soil samples from each chosen house yard and empty lot were collected and 20 for school grounds. Empty lots referred to here were abandoned lots that may be frequented by feral animals and children as playground, while sampling sites for school grounds included classroom premises, playground, and gardens. Approximately 200 g soil samples were taken at two different depths: depth 1 at 0–5 cm and depth 2 at 6–10 cm. The soil samples were placed and sealed in polyethylene bags prior to laboratory analysis. Soil texture (e.g., sandy, clayey, silty, or loamy), pH, temperature, and moisture content were determined following standard procedures.

### 2.2. Soil Processing for Isolation and Identification of Soil-Transmitted Helminth Eggs

The soil samples were air-dried overnight and strained in a 125 *μ*m sieve until 2 g of sample filtrate was obtained. The two-gram sample obtained was placed in a calibrated tube and then washed with 0.5% Tween solution. Modified sucrose floatation technique was employed to harvest the eggs from the soil samples [6]. Briefly, 2 g soil sample was washed with 0.5% Tween solution; then the filtrate was washed again with 6 mL distilled water. The suspension was mixed using a mechanical mixer and then centrifuged for 10 minutes at 1800 rpm. After centrifugation, the supernatant was decanted from the tube; then the sediment was mixed with 8 mL of 1.2 specific gravity sucrose solution. The suspension was again mixed thoroughly with a mixer, lifting the bottom to avoid build-up. The tube was centrifuged again for 10 minutes at 1800 rpm. After centrifugation, 1.3 specific gravity of sucrose solution was slowly added into the tube, using a 10 mL syringe, up to the brim. A cover slip was placed on the mouth of the calibrated tube to collect the topmost portion of the sucrose suspension. After which, the cover slip was placed on top of a glass slide with proper label. The slides were observed for presence of STH eggs using compound light microscope at 100x and 400x magnification. The size of the specimens was determined using an ocular micrometer.

### 2.3. Soil Physicochemical Parameters

In obtaining the temperature of the soil sample, a mercury thermometer was placed inside a 5 cm hole for 2 minutes in each collection site. Soil pH was obtained using a pH meter that was placed in a soil-water (2 g : 1 mL) paste for 2 minutes [7]. On the other hand, soil moisture content was determined by gravimetric method using the formula

(1) $$\begin{array}{c} \text{Moisture}\;\text{content}=\frac{w_{{}^{\circ}}-w_{1}}{w_{{}^{\circ}}}\times 100, \end{array}$$

where *w*_°_ is the predried weight of the sample (100 g) and *w*_1_ is the weight after the sample was oven-dried for 24 hours at 125°C.

Lastly, the soil texture (sandy, loamy, clayey, and silty) was estimated following Lesikar method [8]. Briefly, water was added to the soil at 1 mL : 2 g proportion. Then the soil was kneaded until bolus was formed.

### 2.4. Data Analysis

Chi-square test was used, at *α* = 0.05, for the comparison of prevalence. For the comparison of density of eggs among the soil samples from different locations and among soil textures Kruskal-Wallis *H* test was used. For the comparison of mean intensities of eggs between depths, Mann-Whitney *U* Test was used. Relationship between contamination rates and soil physicochemical properties was analyzed using Pearson's correlation analysis.

## 3. Results

Studies on soil-transmitted helminth (STH) mostly focused on the extent of its infection in humans, particularly among children. However, STH eggs are first released and incubated in soil before infecting the hosts. Hence, soil is considered as the greatest source of STH infection. However, there are relatively few studies in the Philippines conducted to determine the extent of STH eggs contamination in soil, particularly* Toxocara* sp. eggs.

**Figure 1:**
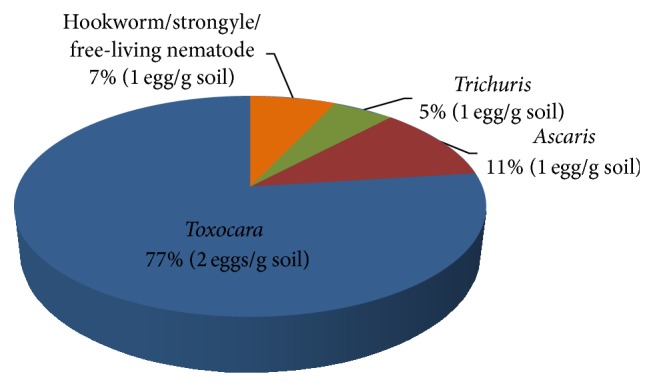
Prevalence (%) and mean density of soil-transmitted helminth (STH) eggs collected from soils in selected towns of Laguna, Philippines.

The present study revealed that, of the 1480 samples collected, 460 (31%) were found positive for STH eggs.* Toxocara *showed the highest prevalence rate of 77%, followed by* Ascaris* (11%), hookworms/strongyles/free-living nematodes (7%), and* Trichuris* (5%) (Figure 1). The eggs and/or larvae of hookworms, strongyles, and free-living nonparasitic nematodes were grouped together as they were difficult to distinguish from one another. Meanwhile, only* Toxocara* prevalence and density in soil were considered in the study for comparison among various sampling sites since it showed a remarkably high prevalence. The prevalence rate for* Toxocara* eggs in Bay, Calauan, Los Baños, and Calamba was 28%, 26%, 33%, and 38%, respectively. The prevalence of* Toxocara* eggs in Calamba and Los Baños (urban) was relatively higher than in Calauan and Bay (rural). However, the difference was not significantly different among them (*X* ^2^ = 1.503, d.f. = 3, *P* = 0.373). The mean egg density for Calamba was 2 eggs/g soil and 1 egg/g soil for the three towns.

**Figure 2:**
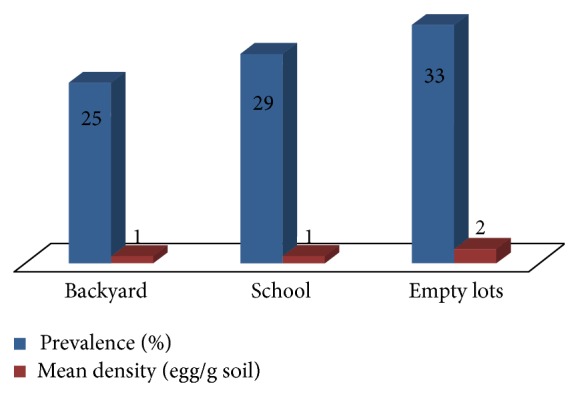
Prevalence and mean density of* Toxocara* eggs in soils from various sampling sites of selected towns in Laguna, Philippines.

On the other hand, assessment of the prevalence and mean density of* Toxocara* egg contamination in soils from schools, empty lots, and house yards was also done in the study (Figure 2). Samples were obtained from these specific locations where children usually visit to play. Results showed that* Toxocara* eggs were more prevalent in empty lots (33%) followed by schools (29%) and backyards (25%). However, prevalence among the various sampling sites was not significantly different (*X* ^2^ = 1.627, d.f. = 2, *P* = 0.546). The mean egg density for empty lots was 2 eggs/g soil while the rest had 1 egg/g soil.

**Figure 3:**
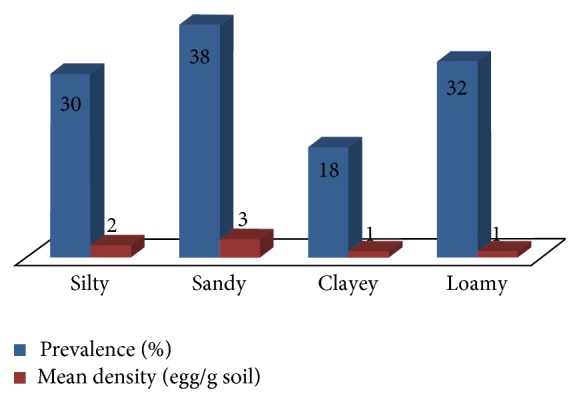
Prevalence and mean density of* Toxocara* eggs in different soil textures from selected towns in Laguna, Philippines.

Development of STH eggs in the outside environment, particularly in soil, is affected by several factors such as temperature, humidity, pH, depth, and soil texture [9–12]. These might affect their development by hastening their embryonation, viability, infectivity, and size. Hence, the correlation between soil physicochemical factors such as soil texture, temperature, moisture content, pH, and depth on the prevalence and mean density of* Toxocara* eggs was also assessed in this study.  Figure 3 shows the prevalence of* Toxocara* eggs among different soil textures. It was observed that sandy soils had the highest prevalence (38%), followed by loamy (32%), silty (30%), and clayey soil (18%). However, there was no statistical difference between the prevalence of eggs in different soil textures (*X* ^2^ = 1.736; *P* = 0.359). In terms of density, sandy soil also harbored the most number of STH eggs (3 eggs/g soil) while clayey soil had the least (1 egg/g soil).  Table 1 shows the mean temperature, moisture content, and pH of the soils from each village in the four towns of Laguna, while Table 2 shows the correlation coefficient (*r*) values to determine the relationship of the environmental factors on the prevalence and mean density of eggs. Results revealed positive correlations between prevalence and mean density with all the physicochemical factors mentioned above. There is a positive correlation (*r* = 0.680; *P* = 0.152) between temperature and the density of eggs in soils, but a weak positive correlation with prevalence (*r* = 0.343; *P* = 0.405), although both are not statistically significant. There is also a significant strong positive correlation between prevalence and moisture content (*r* = 0.799; *P* = 0.027) and with parasite density (*r* = 0.627; *P* = 0.038). On the other hand, pH showed also a positive but moderate correlation with prevalence (*r* = 0.567; *P* = 0.143) and density of* Toxocara* eggs (*r* = 0.430; *P* = 0.560), although statistically not significant.

**Figure 4:**
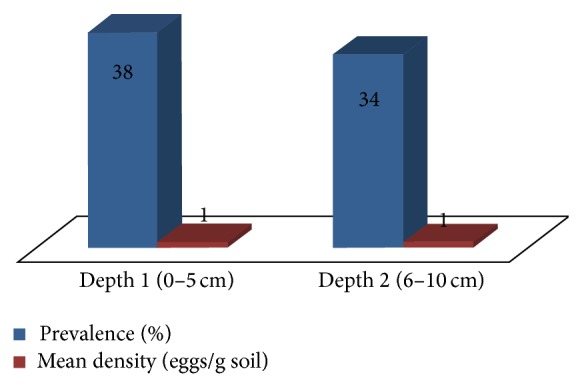
Prevalence and mean density of* Toxocara* eggs at different depths in soils from selected towns in Laguna, Philippines.

Meanwhile, depth 1 (0–5 cm) and depth 2 (6–10 cm) showed prevalence of 38% and 34%, respectively; however, there was no significant difference between the two depths (*U* = 1.361, d.f. = 1, *P* = 0.249). The mean density for both depths was 1 egg/g soil. Depth 1 at 0–5 cm depth showed higher* Toxocara* eggs prevalence (38%) than depth 2 at 6–10 cm depth (34%) (Figure 4); however, this was not significant (*X* ^2^ = 7716; *P* = 0.352). In addition, the mean densities of STH eggs between depth 1 (0–5 cm) and depth 2 (6–10 cm) were both 1 egg/g soil.

## 4. Discussion

Dogs and cats are the definitive hosts of* Toxocara canis *and* Toxocara cati,* respectively. The high prevalence of* Toxocara* eggs in soil could be an evidence of dogs and cats' feces contaminating the soils. This result is also supported by the study done by Tujan and Paller, 2012 [13], which revealed that 22% of household dogs in Calamba were positive for* T. canis.* These infected dogs could be the source of parasite eggs contaminating the soil. It is also known that* Toxocara* can be transmitted via the placenta which increases the chance of pups and kittens becoming infected by* Toxocara.* Hence, this increases the prevalence of this helminth in dogs and cats subsequently increasing the chance of contaminating the soils through indiscriminate defecation.* Toxocara* is also considered as zoonotic parasite; hence, humans are vulnerable to infection.* Ascaris lumbricoides, *hookworms, and* Trichuris trichiura *are also soil-transmitted helminthes, which are infective to humans, with animals and human fecal wastes as source of soil contamination. Also, as observed, some areas of the study sites still do not have latrines or toilets, particularly in slum areas, where people may indiscriminately defecate and contaminate the soils. This condition may even be aggravated during typhoons and floods. Hence, these instances contribute to the contamination of soils with STH eggs and could contribute to their transmission.

STH infection in the Philippines has not yet been eradicated and is still prevalent. In the survey done by the department of health, 2004, 23.2% of Filipinos examined were infected with* A. lumbricoides*. Some of these subjects were also infected with* T. trichiura* and hookworm due to their similar mode of transmission [14]. In addition, Fajutag and Paller [4] also revealed that 43% of public school children in Los Baños showed positive for* Toxocara* antigen detected through ELISA test. Thus, the high prevalence of STH eggs in soil in the study is concomitant with the high prevalence of human infection.

Prevalence of helminth eggs was also compared between urban and rural areas. Although not significant, the higher prevalence of helminth eggs in soils from urban areas may be due to high population density, which increases the possibility of soils to be contaminated with helminth eggs by animal and human wastes. The presence of stray dogs and cats that may indiscriminately defecate in the area also contributes to soil contamination with these parasites' eggs. Mizgajska-Wiktor and Jarosz [15] also compared prevalence of* T. cati *and* T. canis* eggs between rural and urban areas in Poland. They obtained 19.8% and 15.6% positive samples in urban and rural areas, respectively. Similar results were obtained by the study of Rai et al. [16]. Furthermore,* Ascaris *sp. eggs were reported to be more prevalent in soils from urban areas while hookworm larvae are more numerous in soils from rural areas [17].

On the other hand, the high prevalence and mean density of* Toxocara* eggs in empty lots may be due to lack of fences or walls which would allow infected animals to freely defecate and contaminate the soils with parasites. Schools, on the other hand, in all sampling sites were secured by tall walls or gates preventing intruders, including animals, to enter the school parameters, hence the low reported prevalence and density of STH eggs. Medina and Paller [18] had similar findings where STH eggs were most prevalent in sandy soils and least prevalent in clayey soils. Brown [10] reported that clayey soils do not provide enough oxygen needed for STH egg development. Furthermore, sandy and loamy soils are preferred by animals for defecation as they have loose texture for animals to burrow their feces deeper into the soil. Also, STH eggs survive more in sandy soils, with high silt content, since they provide aeration and moisture for their development [17–19]. Moreover, results revealed that STH eggs were also prevalent in basic soil conditions. The present study suggested that* Toxocara* eggs were more prevalent in conditions of high temperature and high moisture content and basic soil condition. This study revealed that soil conditions in some areas in Laguna provide optimum condition for development and embryonation of STH eggs. Soil conditions in Laguna with mean temperature (31.54 ± 2.76°C), mean moisture content (37 ± 6.02%), and mean pH (7.3 ± 0.27) are typical tropical conditions that further allow development and transmission of the STH parasites. The result of this study is supported by the work of Lesikar et al. [8] where they identified that the optimum temperature for the embryonation of STH eggs ranges from 21°C to 28°C. Hotez [19] also claimed that 20°C–30°C is suitable for hookworm eggs. In the study conducted by Arene [20] the most suitable temperature for* Ascaris suum *is between 16 ± 1°C and 34 ± 1°C; as the temperature increases within this range, the development of the egg is hastened. This might be due to the effect of heat to chemical reactions occurring inside the egg for its development. Enzymes might be activated easily when there is a higher temperature and molecules come in contact more often when excited due to the energy from heat. This is also evident in the observation of* Ascaris* eggs in freezing temperatures, which halts development; this is mainly due to the inhibition of the chemical reactions needed for the egg development [20]. However, the development is only postponed but will continue after incubation at an optimum temperature, which means that* Ascaris* eggs are still viable after winter.

Moisture is needed by STH eggs to prevent desiccation or dehydration and hookworm filariform larvae especially need a moist environment since they are very sensitive to sunlight and dry condition. The moisture in the environment is also used by the hookworm larvae for moving [19]. This is related to the increased prevalence of STH eggs during the rainy season which is already observed by a number of studies [16, 21]. Moisture in soils could also provide egg with the ions needed for egg development. Some reports mentioned that helminth eggs are said to tolerate a large range of pH. However,* Ascaris suum *eggs were said to have an arrested development when placed in an acidic environment. Hookworms, on the other hand, tolerate pH range of 4.6–9.4 and will still be able to hatch and infect [22]. According to Hotez et al. [17]* Ascaris* sp. eggs increase survivability by increasing soil depth. Deeper soils provide STH eggs protection from direct sunlight and provide higher moisture [11]. Thus, soil depth until 10 cm may still be infested with STH eggs. However, it might be interesting to examine for future studies the presence of STH eggs in depths deeper than 10 cm.

## 5. Conclusion

The present study revealed that the contamination of soil with fecal matter is ubiquitous. In addition, the prevalence of soil-transmitted helminthes with zoonotic potential, particularly* Toxocara* sp. eggs, in Laguna, could be considered relatively high (31%) and thus alarming. The zoonotic potential of* Toxocara *is underestimated compared to other STH parasites such as* Ascaris*. However, in this study,* Toxocara *sp. eggs showed the most prevalent STH eggs harvested from the soils and thus posing zoonotic infection risks. Local executives, therefore, should go for strict implementation of local ordinances regulating the care and custody of dogs and cats, including regular deworming of pets and other domestic animals. It is also important to emphasize to the children the importance of washing hands especially after playing with soil. As parasitic contamination in soils is a public health issue, this data should be helpful in the development of appropriate education, control, and prevention strategies of this parasitosis.

It is recommended that data will be gathered also in various parts of the country which could be subdivided into groups according to geographical location, ecological zone, socioeconomic designation, and season (wet and dry).

## Figures and Tables

**Table 1:** Some physicochemical characteristics of soils from selected towns in Laguna, Philippines.

Municipality		Mean	
	Temperature (°C)	pH	Moisture (%)
Bay			
San Isidro	30.0 ± 0.00	6.8 ± 0.43	15.30 ± 0.60
Tagumpay	28.5 ± 0.38	6.8 ± 0.58	5.30 ± 7.88
Tranca	28.9 ± 1.23	6.6 ± 0.29	20.0 ± 11.05
Calauan			
Perez	30.14 ± 0.69	7.2 ± 0.24	82.2 ± 5.313
Lamot II	26.86 ± 1.07	7.5 ± 0.18	82.5 ± 4.76
Imok	27.14 ± 1.07	7.4 ± 0.24	77.5 ± 3.85
Los Baños			
Bambang	27.9 ± 1.86	7.1 ± 0.38	12.2 ± 7.64
Batong Malake	30.5 ± 2.31	7.1 ± 0.47	18.0 ± 10.01
Bayog	28.0 ± 0.81	7.5 ± 0.40	13.5 ± 9.27
Maahas	27.4 ± 0.94	7.4 ± 0.41	10.2 ± 8.40
Mayondon	28.9 ± 1.07	6.3 ± 0.69	14.8 ± 8.47
Calamba			
Lecheria	30.3 ± 2.19	7.2 ± 0.28	15.9 ± 6.70
Looc	34.3 ± 4.79	7.3 ± 0.18	11.4 ± 8.10
Uwisan	35.0 ± 2.53	7.4 ± 0.39	13.9 ± 6.39
Singko	31.0 ± 3.45	7.2 ± 0.30	12.5 ± 7.02
La Mesa	33.8 ± 2.89	7.3 ± 0.32	15.6 ± 6.71
Bucal	31.1 ± 4.05	7.3 ± 0.27	15.0 ± 6.98

**Table 2:** Correlation coefficient (*r*) of the prevalence and density of STH eggs with some physicochemical parameters of soils from selected towns in Laguna, Philippines.

Municipality	Temperature (°C)	pH	Moisture (%)
Bay	0.204	0.500	0.966
Prevalence	0.706	0.950	0.500
Density			
Calauan			
Prevalence	0.495	0.700	0.546
Density	0.565	0.756	0.799
Los Baños			
Prevalence	−0.485	0.742	0.574
Density	0.522	0.380	0.676
Calamba			
Prevalence	0.196	−0.674	0.326
Density	0.475	0.239	0.215
All sites			
Prevalence	** 0.343**	** 0.567**	**0.799** ∗
Density	** 0.680**	** 0.431**	**0.627** ∗
*P* value			
Prevalence	** 0.405**	** 0.143**	**0.027**
Density	** 0.152**	** 0.560**	**0.038**

^*^Significant at *P* < 0.05.
